# Have Preferences of Girls Changed Almost 3 Years after the Much Debated Start of the HPV Vaccination Program in the Netherlands? A Discrete Choice Experiment

**DOI:** 10.1371/journal.pone.0104772

**Published:** 2014-08-19

**Authors:** Robine Hofman, Esther W. de Bekker-Grob, Jan Hendrik Richardus, Harry J. de Koning, Marjolein van Ballegooijen, Ida J. Korfage

**Affiliations:** 1 Department of Public Health, Erasmus MC - University Medical Centre Rotterdam, Rotterdam, the Netherlands; 2 Municipal Public Health Service Rotterdam-Rijnmond, Rotterdam, the Netherlands; University of Missouri Kansas CIty School of Medicine, United States of America

## Abstract

**Objectives:**

To assess how girls' preferences have changed almost 3 years after the much debated start of the human papillomavirus (HPV) vaccination program.

**Methods:**

A discrete choice experiment (DCE) was conducted among girls aged 11–15 years who were invited, or were not yet invited, to get vaccinated. A panel latent class model was used to determine girls' preferences for vaccination based on five characteristics: degree of protection against cervical cancer; duration of protection; risk of mild side-effects; age of vaccination; and the number of required doses of the vaccine.

**Results:**

The response rate was 85% (500/592). Most girls preferred vaccination at age 14 years (instead of at age 9 years) and a 2-dose scheme (instead of the current 3-dose scheme). Girls were willing to trade-off 7% (CI: 3.2% to 10.8%) of the degree of protection to have 10% less risk of mild side-effects, and 4% (CI: 1.2% to 5.9%) to receive 2 doses instead of 3 doses. Latent class analyses showed that there was preference heterogeneity among girls, i.e., higher educated girls and HPV vaccinated girls had a higher probability to opt for HPV vaccination at a higher age than lower educated girls or non-vaccinated girls.

**Conclusions:**

Three years after the start of HPV vaccination program the risk of mild side-effects and age at vaccination seem to have become less important. For the Dutch national immunization program, we recommend *not* to lower the current target age of 12 years. A 2-dose scheme may result in a higher uptake and we recommend that if this scheme is introduced, it needs to receive adequate publicity.

## Introduction

Human Papillomavirus (HPV) infection is a necessary factor in the development of cervical cancer [1], [2]. HPV types 16 and 18 are responsible for about 70% of all cervical cancers worldwide [3]. Preferably the HPV vaccine (which protects against those two types) is given prior to the initiation of sexual activity, because the degree of protection is reduced after HPV infection [4]–[6].

Many Western countries have included HPV vaccination in their immunization program. For example, the United Kingdom, Canada, Australia and the Netherlands offer the HPV vaccine to girls at an age between 11 and 14 years; in these countries, the uptake rates range from 50–80%. The willingness to accept HPV vaccination can largely be influenced by general preferences for healthcare interventions [7]. One way to assess preferences is to conduct a discrete choice experiment (DCE), in which people trade off risks and benefits among competing programs [8]. In the design of a DCE it is assumed that a healthcare intervention can be described by its characteristics (attributes) and that the levels of those attributes determine preferences for an intervention [9]. By offering a series of choices between two or more intervention alternatives with different combinations of attribute levels, the relative importance of attributes can be assessed [10]. Previous DCE studies about preferences for HPV vaccination showed that attributes such as the duration and degree of protection against cervical cancer were important among mothers of eligible girls [11], adults from the general public [12], and eligible girls [13].

In the Netherlands, the bivalent HPV vaccine is offered free of costs to 12-year-old girls by sending a personal invitation. These girls do not need their parents' permission when deciding about uptake. Since the introduction of the vaccine in the Netherlands in 2009, uptake rates increased from 52% in 2009 [14] to 59% in 2011 [15]. The introduction of the program coincided with an intensive societal debate involving politics, physicians, media, parents and girls, which may have resulted in uptake rates being lower than expected beforehand. During that period we carried out a DCE to assess girls' preferences for HPV vaccination [13]. We showed how girls made trade-offs between the degree of protection against cervical cancer, the duration of protection, the risk of serious side-effects (e.g. hospitalization), the risk of mild side-effects (e.g. nausea), and age of vaccination. Currently, almost 3 years later, although no serious side-effects have been linked to the vaccine, this has not resulted in a large increase in the vaccination rates.

Therefore, the present study assesses which attributes of HPV vaccination have influenced preferences for HPV vaccination uptake *after* the media debates have ended and in the absence of reports of serious side-effects. To our knowledge, this is the first study to compare preferences for HPV vaccination as expressed in DCEs. We will look at the differences in preferences as measured in 2009 versus 2011. This comparison may provide insight into girls' motivation to be vaccinated or not, how this motivation can change over time, and how to improve dissemination of information about the vaccine.

## Methods

### Attributes and attribute levels

The selection of HPV vaccination attributes and their levels was based on our previous study [13]. However, for the present study we excluded the attribute ‘risk of serious side-effects’ from the choice sets since no serious side-effects of the vaccine have been reported since its introduction in vaccination campaigns. The Centers for Disease Control and Prevention (CDC) state the following about the bivalent vaccine: the bivalent vaccine is safe, it has been in use around the world for several years and has been very safe. However, any medicine can potentially cause a serious problem, such as a severe allergic reaction. The risk of a vaccine causing a serious injury, or death, is extremely small. Life-threatening allergic reactions from vaccines are very rare [16]. Instead, we mentioned in the questionnaire that the risk of serious side-effects on the long term is unknown. We added the attribute ‘number of doses of the vaccine’, because less than the currently applied number of 3 doses is also likely to be effective [17].

The final set consisted of the following attributes: 1) the degree of protection against cervical cancer; 2) the duration of protection; 3) the risk of mild side-effects; 4) the age of vaccination; and 5) the number of doses of the vaccine (Table 1). The levels we used for degree of protection were 50%, 70% and 90%. It is assumed that the protection against cervical cancer is 70%, but since it takes 10 to 15 years for cervical cancer to develop it is not sure yet whether the protection indeed will be 70%. It might also be possible that the protection is lower or a new HPV vaccine will be available in the future that has an effectiveness of 90% [18]. Since to date, follow-up data on HPV vaccinated young women are available for 8.4 years, it is known that protection lasts at least 8 years, but it is unknown how long the duration of protection will be [19]. We therefore wanted to know girls' preferences for a duration of 8 years, 25 years and lifelong protection. The levels of the risk of mild side-effects were 1∶30, 10∶30 and 20∶30, which were based on figures from the Centers for Disease Control and Prevention [20]. We choose 30 as the denominator, because many classes consist of 30 students and therefore girls could interpret the risk as 1, 10 or 20 students in their class suffering from mild side-effects. The side-effects were defined as: pain, itch, redness and swelling on the injection area; fever; headache; dizziness; nausea and fainting within 2 hours after vaccination. The risk of mild side-effects is not modifiable, but if for example girls put a lot of weight on this risk, information about the risk may highlight the short duration of the side-effects. Levels of the age of vaccination were 9, 12 and 14 years. If most girls will have a preference for 9 or 14 years instead of the current 12 years, it might be a possibility to broaden the age range at which girls are offered the vaccine for free. The levels of the number of doses of the vaccine were 2 and 3 doses. If for example most girls have a preference for 2 doses, then uptake may increase if 2 doses are used instead of 3.

**Table 1:** Attributes and levels for HPV vaccination included in the discrete choice experiment design.

Attributes	Levels
Degree of protection against cervical cancer (%)	50, 70, 90
Duration of protection (years)	8, 25, lifetime
Risk of mild side-effects	1∶30, 10∶30, 20∶30
Age at vaccination (years)	9, 12, 14
Number of doses of the vaccine	2, 3

### Study design

The combination of four attributes with three levels each, and one attribute with two levels, resulted in 162 (3^4^×2^1^) hypothetical HPV vaccination alternatives. We generated a subsample of these alternatives using priors available from De Bekker-Grob et al. [13] and a zero prior for the attribute ‘number of doses’ to generate an efficient design by maximizing D-efficiency (using Ngene software, version 1.1.1, http://www.choice-metrics.com/) [21]. Sixteen choice sets were constructed to be able to estimate all main effects. Choice sets consisted of two HPV vaccination alternatives and a ‘no HPV vaccination’ option to allow respondents to ‘opt out’ (Table 2).

**Table 2:** Choice set example.

Attributes	Program A	Program B	No vaccination
Degree of protection against cervical cancer	70%	90%	0%
Duration of protection	Lifetime	8 years	n.a.
Risk of mild side-effects	10∶30	20∶30	No risk
Age at vaccination	12 years	12 years	n.a.
Number of doses of the vaccine	3	3	0
**Which vaccination program do you prefer?**	**A**	**B**	**None**

n.a. = not applicable.

### Study sample

Calculation of the optimal sample size for estimating discrete choice models from DCE data is complicated, as it depends on the true values of the unknown parameters estimated in the choice models [22]. Earlier studies have shown that sample sizes of 300–400 respondents are sufficient for reliable statistical analyses [23], [24]. Therefore, first, we strived to collect at least 400 completed questionnaires. In order to do so, taking into account an expected response rate of at least 80% [13], we recruited a representative sample of n = 592 girls aged 11–15 years through four secondary schools in urban and rural areas in the Netherlands. Second, we checked a posteriori whether our sample size would be sufficient to find significant differences for each attribute (level) at a 5% level using the true values of the estimated parameters and NGene software (http://www.choice-metrics.com/).

### Questionnaire

The first page of the questionnaire provided basic information about HPV vaccination. Next, respondents were asked to indicate per choice set which option appealed to them most. Pictographs were used to illustrate the percentages of the degree of protection and the risk of mild side-effects.

To assess respondents' understanding of the DCE task we included a dominant choice set as a rationality test. In this choice set age of vaccine administration was similar in both alternatives, while one alternative was characterized by logically preferable levels on all other attributes. Also we included four items on a 5-point Likert scale to evaluate whether respondents considered the DCE questions ‘clear-unclear, ‘difficult-easy’, ‘annoying-pleasant’, and the number of questions as ‘too many-not too many’. Convergent validity was checked by asking the respondents to rank the five attributes of HPV vaccination from most important to least important. This ranking is compared with the trade-offs respondents were willing to make between the degree of protection and the other attributes.

The questionnaire used in our 2009 study was pilot tested to check for face validity and for problems in interpretation (n = 16). Because the number of attributes are the same as in the present study and only the attribute ‘risk of serious side-effects’ is replaced with ‘number of doses of the vaccine’, we did not expect problems in interpretation and therefore did not pilot test the questionnaire of the present study.

### Procedure

Respondents completed the questionnaire in the classroom or auditorium during school time. First, general information was given about HPV and vaccination and about the way DCE questions should be completed. Completion of the written questionnaire lasted about 20–30 min. Questionnaires were completed in November and December 2011.

Beforehand, girls' parents had received an information letter covering the purpose, the voluntary nature and anonymity of the study, and an opt-out form. Parents that did not want their daughter to participate could sign the opt-out form. Girls' parents who approved participation did not have to sign an informed consent form. The Medical Ethics Committee of Erasmus MC, University Medical Center Rotterdam declared that this research (number MEC 2011-059) did not fell under the Medical Research Involving Human Subjects Act, because Participants were not subject to procedures or are required to follow rules of behavior.

### Statistical analyses

The DCE was analyzed by taking each choice among the three options (two HPV vaccination options, and a ‘no vaccination option’) as an observation. The utility for “no vaccination” was normalized to zero: V(no vaccination) = 0. Using NLogit software (http://www.limdep.com/), the observations were analysed by a panel latent class model [25]. This model can be used to identify classes in the population, i.e., identifying different utility (preference) functions across unobserved subgroups. Class membership is latent in that each respondent belongs to each class up to a modelled probability and is not deterministically assigned by the analyst a priori. The model is flexible in that the probability that sampled respondents belong to a particular class can be linked to covariates (such as age, education, etc.), hence allowing for some understanding as to the make-up of the various class segments [26]. *Panel* latent class model means that the model accounts for the pseudo panel nature of the DCE data since each respondent completed 16 choice tasks. To determine the number of classes to impose on the model structure, we selected the model with the best fit based on the Akaike information criterion (AIC) [25].

We tested a number of different specifications for the utility (preference) function. After testing for linear continuous effects of the attributes, the following final specification of the utility model was estimated:
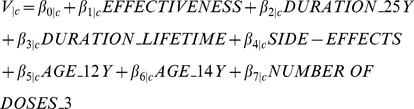
(1)


*V_|c_* represents the observable utility (preference score) that respondents belonging to class segment c have for an HPV vaccination. *β*_1|c−*7|c*_ are class specific coefficients of the attributes indicating the relative weight individuals place on a certain attribute (level). The unobserved component, 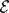, is assumed to be independently and identically (IID) extreme value type 1 (EV1) distributed. In addition to the utility function, the final model allowed for several significant covariates (‘respondent's history of HPV vaccination’ and ‘education’) to enter into the class assignment model. Effects coded variables were used for protection of duration, age at vaccination, and doses of the vaccine. Degree of protection and risk of mild side-effects were coded as a linear term.

The statistical significance of a coefficient (p-value ≤0.05) indicates that conditional to belonging to a class, respondents differentiated between one attribute (or attribute level) and another in making stated choices about HPV vaccination programs. A priori, we expected all attributes to be significant. The sign of a coefficient reflects whether the attribute has a positive or negative effect on the preference score (utility). We expected that the attributes ‘risk of mild-effects’ and ‘the number of doses of the vaccine’ would have a negative effect. The value of each coefficient represents the relative importance respondents assign to an attribute level. Sensitivity analyses were conducted to explore the impact of excluding respondents who failed the rationality test by excluding their data from the sample and re-running the analysis [27], [28].

In terms of the class assignment parameters, statistically significant parameter estimates indicate that the associate covariate (i.e. ‘respondent's history of HPV vaccination’ and ‘education’) can be used to help in understanding the different segments. For example, if the education parameter associated with a particular class in the assignment model is positive and significant, then this is indicative that people who have a higher educational level are more likely to belong to that particular class and, hence, have preferences associated with the utility function belonging to that class as given in Equation (1).

The trade-offs respondents were willing to make between the attributes were calculated by the ratios of the coefficients of the different attributes with the degree of protection as the denominator. These trade-offs were weighted by the probability that a respondent belongs to a given class. Confidence intervals were calculated in Excel using the Krinsky and Robb method [29]. The number of simulations was 65,000 (i.e., 130 Sobol draws×500 respondents).

Since our 2009 study is a point of reference for this study, we compared the similarity of the present sample to the 2009 sample. Mann-Whitney U tests were used for continuous variables and Chi-square tests were used for categorical variables.

## Results

### Respondents

The response rate was 85% (500/592). The mean age of the respondents was 12.9 years; most had a higher level of secondary education (38%) and no religious affiliation (68%). Of the respondents, 63% had already been invited to get vaccinated against HPV of whom 70% had opted for vaccination (Table 3). Compared to the 2009 sample, respondents in the present sample were younger (difference 0.4 years, *p*-value<0.01); more respondents had a lower or intermediate educational level and less girls had a higher educational level (*p*-value<0.01); and less respondents were vaccinated against HPV (*p*-value = 0.045).

**Table 3:** Characteristics of the study respondents (n = 500).

Characteristics		
	Mean	(SD)
**Age** (years)	12.9	(0.96)
range	11–15	
	**N**	**(%)**
**Educational level**		
Low	145	(29.1)
Intermediate	164	(32.9)
High	189	(38.0)
**Religion**		
None	338	(68.0)
Christian	124	(24.9)
Muslim	28	(5.6)
Other	7	(1.4)
**Country of birth**		
The Netherlands	472	(99.0)
**Country of birth of parents**		
Both parents in the Netherlands	385	(79.9)
One parent outside the Netherlands	42	(8.7)
Both parents outside the Netherlands	55	(11.4)
**HPV vaccination**		
Invited to get vaccinated against HPV	311	(62.7)
HPV vaccinated	220	(70.7)
Intention if not yet invited:		
Low	20	(10.9)
Neutral	31	(16.8)
High	133	(72.3)

### DCE results

Based on the AIC criterion, three classes were identified (Table 4). The average class probabilities within the sampled population were 31.0%, 45.5% and 23.5% for latent class 1, 2 and 3, respectively. The probability to belong to a specific class depended on the respondent's level of secondary education and whether she has been vaccinated against HPV. Namely, girls attending higher levels of secondary education and HPV vaccinated girls had a higher chance to belong to latent class 3, than lower educated and non HPV vaccinated girls(Dutch secondary schools have different educational levels). Respondents belonging to latent class 3 preferred vaccination at age 12 years to age 9 years, which was not a significant preference for respondents who belong to latent class 1 and 2. Most of the estimated coefficients for each latent class had the expected sign and were significant in most cases (Table 4). Although all five HPV vaccination attributes significantly influenced girls' preferences, the preference heterogeneity was substantial. Respondents in all classes preferred a lower risk of mild side-effects and a higher degree of protection to a higher risk and a lower degree of protection; they also preferred 25 years of protection to 8 years of protection. Respondents belonging to class 1 preferred 25 years of protection to 8 years of protection, rather than lifetime protection to 8 years of protection. Respondents who belong to latent class 2 and 3 preferred 2 doses to 3 doses, and preferred vaccination at 14 years rather than at 9 years, whereas respondents belonging to latent class 1 showed no significant preference for the number of doses or the age of vaccination. Sensitivity analyses showed that excluding the data of ten out of 500 respondents (2%) who ‘failed’ the rationality test had no relevant impact on the size or relative importance of the attributes.

**Table 4:** Respondents' preferences for HPV vaccination based on a panel latent class model.

	Latent Class 1		Latent Class 2		Latent Class 3	
Attribute	Coeff.		Coeff.		Coeff.	
**Risk of mild side-effects (per 10%)**	−0.49	***	−0.41	***	−0.30	***
**Degree of protection against cervical cancer (per 10%)**	1.33	***	0.40	***	0.73	***
**Duration of protection:**						
8 years (reference)	−0.50		−0.81		−0.89	
25 years	0.84	***	0.29	***	−0.19	***
Lifetime	−0.34	**	0.52	***	1.07	***
**Age at vaccination:**						
9 years (reference)	0.05		−0.29		−0.32	
12 years	−0.12		−0.01		0.16	***
14 years	0.07		0.30	***	0.16	***
**Number of doses of the vaccine:**						
2 doses (reference)	0.14		0.10		0.08	
3 doses	−0.14	*	−0.10	***	−0.08	**
**Constant**	−4.39	***	1.73	***	−4.98	***
	**Class probability model**					
Constant	−0.0851		0.3705	**	-	
Higher eduction	−0.0007	**	−0.0005	*	-	
Vaccinated	−0.0005	*	−0.0005	*	-	
	**Class probability (%)**					
Average class probability	31.0		45.5		23.5	
	**Model fits**					
Log-likelihood	−4.545.47					
Pseudo R-squared	0.481					

Notes: (1) ***, **, * denotes significance at 1%, 5%, and 10%, respectively; (2) Effects coded variables used for protection duration, age at vaccination, and doses of the vaccine; (3) Coeff. = coefficient; (4) number of observations = 7,976.

### Trade-offs

Overall, respondents were willing to trade-off 7% (CI: 3.2% to 10.8%) of the degree of protection to have a 10% less risk of mild side-effects. To obtain protection against HPV for 25 years instead of 8 years, they were willing to trade-off 18% (CI: 8.6% to 29.6%), and to obtain lifetime protection instead of 8 years of protection, they were willing to trade-off 21% (CI: −0.1% to 37.2%). Respondents were willing to trade-off 4% (CI: 1.2% to 5.9%) to receive 2 doses instead of 3 doses. To get a vaccination at age 12 or 14 years, instead of at 9 years, respondents were willing to trade-off 4% (CI: −2.4% to 8.6%) and 8% (CI: −0.6% to 16.7%), respectively (Table 5).

**Table 5:** Respondents' trade-offs between degree of protection versus various aspects of a vaccination program as used in the present study.

Change in levels	Willingness to trade degree of protection	
	%	(CI)
Per 10% less **risk of mild side-effects**	6.7	(3.2 to 10.8)
A **protection duration** of 25 years instead of 8 years	17.8	(8.6 to 29.6)
A lifetime **protection** instead of 8 years	21.4	(−0.1 to 37.2)
A vaccination at **age** 12 years instead of 9 years	4.4	(−2.4 to 8.6)
A vaccination at **age** 14 years instead of 9 years	8.2	(−0.6 to 16.7)
A vaccination program consisting of 2 instead of 3 **doses**	3.5	(1.2 to 5.9)

Note: CI = 95% confidence interval based on the Krinsky Robb method adjusted for class probabilities.

### DCE rationality

The dominant choice set was answered correctly by 490/500 (98%) of the respondents; 83 respondents completed the ranking test incorrectly (e.g. giving the same rank to multiple attributes) and were excluded from this ranking analyses. The most important attributes according to the ranking test were: the degree of protection (70%); the duration of protection (17%); the risk of mild side-effects (8%); the number of doses (4%); and the age of vaccination (2%) (n = 407). The trade-offs respondents were willing to make between the degree of protection and the other attributes indicated the following order of importance of attributes: duration of protection, followed by the risk of mild side-effects, the number of doses of the vaccine, and age at vaccination (Table 5). Thus, the ranking test supports the convergent validity of the DCE results.

The mean evaluations of the DCE questions were (range 1–5): ‘unclear-clear’ (M = 3.48, SD = 1.14), ‘difficult-easy’ (M = 3.53, SD = 1.14), ‘annoying-pleasant’ (M = 2.82, SD = 1.01), and ‘too many questions-not too many questions’ (M = 2.56, SD = 0.93).

## Discussion

We used a DCE to determine girls' preferences for HPV vaccination almost 3 years after the much debated start of the HPV vaccination program. Overall, girls were willing to trade-off 18% of the degree of protection to obtain a vaccination with 25 years protection instead of 8 years protection, and trade-off 7% to have a 10% less risk of mild side-effects. To receive 2 doses of the vaccine instead of 3 doses, they were willing to trade-off 4% of the degree of protection. Furthermore, it appeared that higher educated girls and HPV vaccinated girls have a higher probability to opt for HPV vaccination if it is offered at age 12 years instead of at age 9 years, than girls with lower education levels or girls who were not vaccinated.

When comparing these reported trade-offs with those of our previous study in 2009 [13], the changes are not substantial. The risk of mild side-effects became less important: in 2011 girls were willing to trade off 7% of the degree of protection (CI: 3.2% to 10.8%) to obtain a 10% less risk of mild side-effects, while in 2009 they were willing to trade-off 18% (CI: 13.8% to 22.4%). Also, it became less important to obtain lifetime protection instead of 8 years (in 2011) or 6 years protection (in 2009), as this trade-off was no longer significant in 2011 (21%, CI: −0.1% to 37.2%) whereas it was in 2009 (38%, CI 32.1% to 44.3%). Also, age of vaccination at 12 years instead of at 9 years was no longer significant in 2011 (2011: 4%, CI: −2.4% to 8.6%; 2009: 7%, CI: 2.6% to 10.6%).

In summary, almost 3 years after initiation of the HPV vaccination campaign on the Netherlands, the risk of mild side-effects and age at vaccination seem to have become less important. Potentially, the girls had a better idea about which mild side-effects to expect and were less concerned about them. Also, the importance of the degree of protection may have gained value for the girls. The age of vaccination might be less of an issue in 2011 given the longer duration of protection, i.e. 8 years in 2011 compared with 6 years in 2009.

There was preference heterogeneity among the girls, i.e. higher educated girls and HPV vaccinated girls have a higher probability to opt for HPV vaccination if it is offered at age 12 years instead of at age 9 years, than girls with lower education levels or girls who were not vaccinated. Furthermore, the majority of girls (including higher educated girls and HPV vaccinated girls) also preferred vaccination at age 14 years to vaccination at age 9 years. In other words, most girls did not prefer vaccination at the age of 9 years. Overall, girls were willing to trade-off 3.5% of the degree of protection to receive 2 doses instead of 3 doses, and most girls also preferred a 2-dose scheme to the current 3-dose scheme. Recently, the Netherlands National Institute for Public Health and the Environment decided that a 2-dose scheme will be introduced, because 2 doses are found to provide as much protection as 3 doses as long as the vaccination is given before girls turn 15 years of age [30], [31]. Since we showed that girls preferred a 2-dose scheme, this new strategy may result in a higher vaccination uptake. We want to stress that this revised vaccination program needs to receive adequate publicity. Surprisingly, it seems that some girls preferred 25 years of protection to lifetime protection. The concept of ‘lifetime’ might be too vague for these young girls and they may be unable to correctly judge its value; a protection period of 25 years might be interpreted by them as a very long period and it may sound more ‘concrete’.

A strength of the present study is the large number of respondents (n = 500) and the high response rate (85%). A limitation might be that we did not include protection against genital warts as an attribute.

In conclusion, this study shows that, almost 3 years after the much debated start of the HPV vaccination program in the Netherlands, trade-offs that girls are willing to make have not changed substantially. The risk of mild side-effects and age at vaccination still influenced the girls' preferences, but seem to have become less important. This study shows that there was preference heterogeneity among the girls, with higher educated girls and HPV vaccinated girls having a higher probability to opt for HPV vaccination at a higher age, than girls with lower education levels or girls who were not vaccinated. Also, since most of the girls preferred vaccination at age 14 years to vaccination at age 9 years, we recommend not to lower the current target age of 12 years in national immunization program in countries such as the Netherlands, Denmark, Sweden, Norway and United Kingdom. We also recommend to introduce a 2-dose scheme (instead of the current 3-dose scheme), because the girls are far from indifferent to the choice between 2 and 3-dose scheme.
